# Land-use patterns and their implication on malaria transmission in Kilosa District, Tanzania

**DOI:** 10.1186/s40794-018-0066-4

**Published:** 2018-06-20

**Authors:** Phillipo Paul, Richard Y. M. Kangalawe, Leonard E. G. Mboera

**Affiliations:** 1 Institute of Resource Assessment, University of Dar es Salaam, P.O. Box 35097, Dar es Salaam, Tanzania; 2 National Institute for Medical Research, P.O. Box 9653, Dar es Salaam, Tanzania

**Keywords:** Agriculture, Land use patterns, Malaria transmission, Tanzania

## Abstract

**Background:**

Understanding of the land use and malaria transmission among farming communities in Tanzania is of great significance. Water resource development projects, deforestation, wetland cultivation, and land use changes for agricultural purposes all expand habitats for malaria-carrying mosquitoes. The main objective of this study was to assess land use patterns and their implication on malaria transmission in two villages in Kilosa District, Tanzania.

**Methods:**

Multiple research methods were used for data collection, including household interviews using a structured questionnaire; key informant interviews; transect walks and direct field observations. A larval search was conducted using the dipper standard method whereby mosquito larvae and pupae were identified to genus level. Data analysis was undertaken using the Stata software version 10 and descriptive statistics were used.

**Results:**

A total of 211 diverse mosquito breeding habitats were surveyed during this study. The mosquito breeding sites ranged from small areas such as hoof prints and coconut shells to large ones such as swamps created through anthropogenic activities. The relationships between land use patterns and malaria transmission were statistically insignificant, indicating that malaria transmission in Kilosa could possibly be due to other human activities, including seasonal movement to distant farms during farming seasons. Communities were knowledgeable about malaria preventive measures such as the use of mosquito nets. While knowledge that links mosquitoes and malaria was relatively high among respondents, knowledge related to mosquito ecology and breeding sites was generally low.

**Conclusion:**

Although analysis of land use patterns did not show statistical significance in the study area, agricultural activities, brick making and settlement seem to be highly linked to malaria transmission. The association of land use patterns and malaria transmission is well observed in habitats created that harbour mosquitoes, and evidenced by presence of immature Anopheles mosquito larvae. Lack of knowledge of the epidemiology of transmission by the inhabitants is a major issue. Although it might be difficult to change land use patterns, as they are driven by economic necessity, future reduction of spread, through better education, is something that could be modified. In addition, more detailed studies are recommended to further confirm the linkages between land use/cover changes and malaria transmission in the study area.

## Background

Globally there is a significant correlation between land use and malaria transmission. Water resource development, deforestation, wetland cultivation, crop cover and land use changes for agricultural purposes in the highlands, wetland cultivation and increase in urban agriculture all expand habitats for malaria carrying mosquitoes [1–6], resulting in the upsurge of malaria transmission in various places.

Various scholars hold that in Africa malaria is predominantly a rural disease and it has been observed that Anopheles mosquito breeding decreases with urbanization [2, 5, 6]. However, in various cities in Africa with poor environmental management and peri-urban agriculture including fish ponds are providing favourable habitats for mosquitoes [2, 6, 7] thereby enhancing malaria transmission due to higher adult Anopheles densities with more malaria episodes than in non-agricultural locations [5, 8].

In the rural areas, deforestation is one of the most potent factors in emerging and re-emerging infectious diseases [9]. Deforestation is driven by a wide variety of human activities, including agricultural development, logging, transmigration programmes, road construction, mining, and hydropower development [10]. These processes alter the various elements of local ecosystems such as microclimate, soil, and aquatic conditions, and most significantly, the ecology of local flora and fauna, including human disease vectors like Anopheles mosquitoes. As reported by Yasuoka and Levins [9], mosquitoes are very sensitive to environmental changes. Deforestation and land transformations have influence on vector Anopheles, especially larval and adult survivorship, reproduction, and vector capacity, leading to a prolonged seasonal malaria transmission [11, 12]. For example, the re-emergence of malaria in the highlands of Western Kenya has been greatly blamed on the clearing of the forests for the development of tea estates [13]. Similar observations have been reported in the highlands of Tanzania in Muheza, Mpwapwa, Iringa, Rungwe and Muleba districts [14], the Rukungiri and Kabale districts of Southwest Uganda, and in the Rwanda highlands [15–19].

The effects of deforestation on malaria transmission may be spatially variable and highly dependent on vector distribution [20]. This is because the vector species could adapt to different types of land cover and therefore could make the effect on malaria transmission regionally distinctive and even locally specific. These scholars reported, for instance, that the clearing of forest lands for rice cultivation may provide more favourable conditions for the *Anopheles gambiae* or *An. albitarsis* but can reduce transmission in areas where *An. dirus* is the main vector. However, in situations where the forest clearing leads to tree crop plantations, the *An. dirus* may find suitable breeding conditions in the plantations similar to the vector’s natural habitats [20].

Reclamation of wetlands for cultivation in Africa has also been associated with increased vector abundance and malaria transmission [21]. Minakawa [22] reported that extensive cultivation of the valley bottoms across East Africa as a result of rapid population and demand for more food has changed the local ecology. These wetlands were covered with natural papyrus, which limits the breeding of *An. gambiae* due to the dense vegetation and the oily layer. The elimination of the papyrus and the reclamation of the swamps have led to increase in temperatures, promoting the breeding and survival of the mosquitoes and thereby increasing malaria transmission in the affected areas [15, 16]. The Kilombero Valley in Tanzania is one of the areas described as highly malaria-endemic tropical wetland dominated by subsistence agriculture [23].

Changes in land use/cover patterns have also been associated with the emergence of highland malaria. Traditionally most of tropical highlands had little or no malaria [24–28]. This situation has changed with several highland areas in the tropics experiencing major malaria epidemics in the last 30 years [14, 18]. The increasing prevalence of malaria in the highlands of Africa, especially in Eastern Africa, has to a large extent been attributed to agricultural practices that have resulted in changes in rainfall patterns, temperature, and vegetation and ecology that is often accompanied by a massive increase in population in areas of unstable endemic transmission [17]. This study, therefore, assessed the changes in land use patterns and their implication on malaria transmission in Kilosa district of central Tanzania.

## Methods

### The study area

**Fig. 1:**
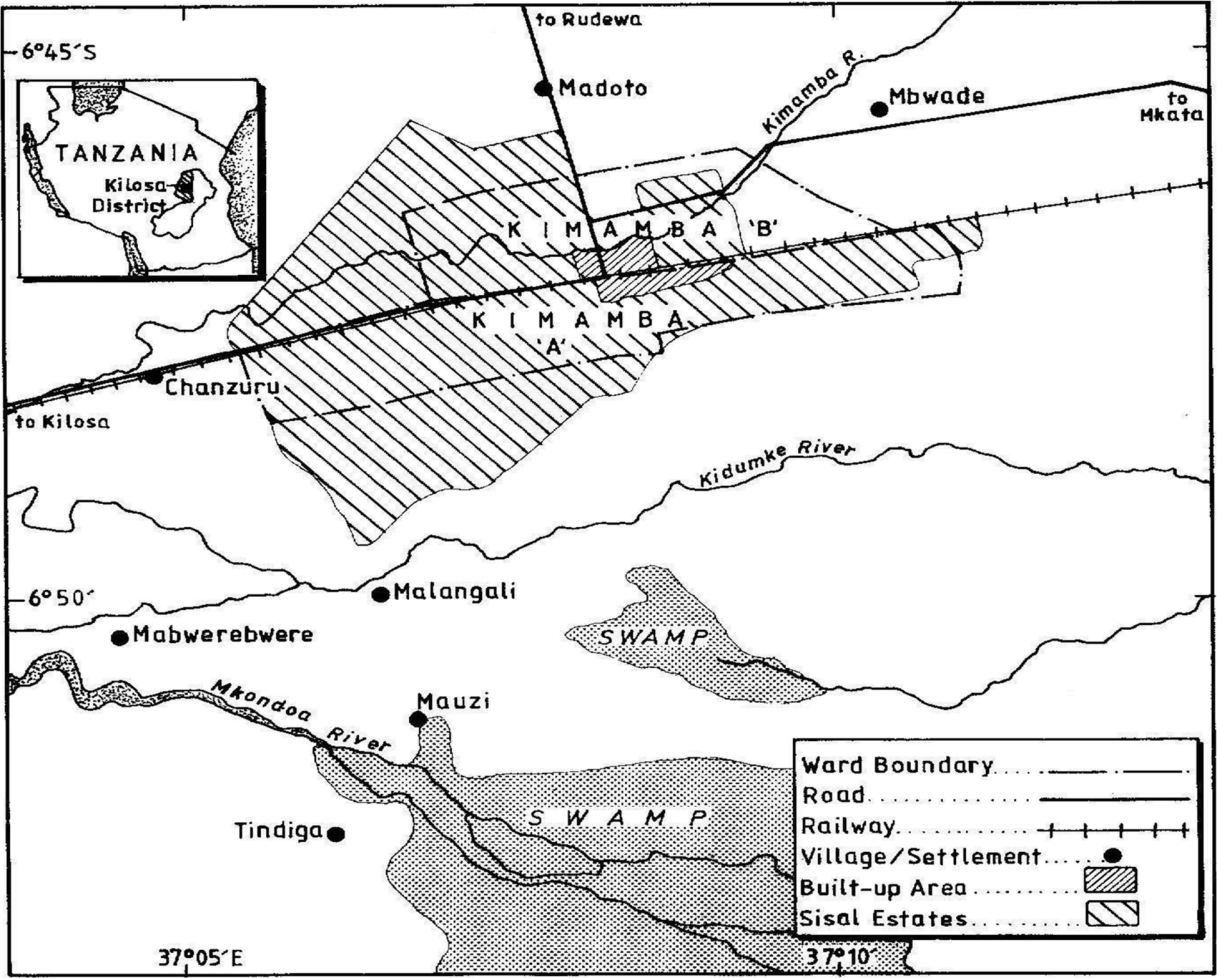
Map of Kimamba villages

This study was conducted in Kimamba “A” and “B” villages in Kimamba ward (Fig. 1) in Kilosa District in central Tanzania. The study villages had a total population of 10,562 persons [29]. The district is located in a tropical semiarid environment with a bimodal type of rainfall and an average temperature of 25 °C [30]. The short rains are received from October through December and the long rains from mid-February through May [30–33]. The climatic condition of the district varies depending on the agro-ecological zones, with the highest parts of the district at 2200 m above sea level getting annual rainfall ranging from 1000 to 1600 mm. This area is characterized by moderately fertile well drained sandy clay loam soils. The central and southern parts experience an average rainfall of 800 – 1400 mm with poorly drained black clays and loamy soils. The dominant land use is agriculture, where maize, paddy, sisal, sugarcane and vegetables are cultivated. The selected study villages were involved in a larger study titled Integrated Research Partnership for Malaria through an Ecohealth Approach in East Africa.

### Research methods

Multiple research methods were used for data collection, including household interviews using a structured questionnaire; key informant interviews; transect walks and direct field observations. Household interviews involved a total sample of 399 households were selected using a simple random sampling technique and representing 10% of households in the study area. The sample comprised of 202 and 197 households in Kimamba “A” and “B” village respectively. The survey questionnaire covered various aspects related to types of land uses and patterns related to malaria transmission, diversities of mosquito breeding sites and control methods employed by the inhabitants. The questionnaire also solicited information on household demographic characteristics, land use information including crop production and animal husbandry, household livelihood and anthropogenic activities associated with malaria transmission, including people’s knowledge, perceptions, and behaviours in relation to malaria transmission and control.

Key informants included various district officials (medical officer, planning officer, lands officer, forest officer, education officer, community development officer, executive officer and water engineer), village leaders and village natural resources committees. The interviews focused on a wide range of information such as types of land use and natural resources management practices in relation to malaria transmission. Field observations involved transect walks that were undertaken following in-depth discussions with key informants. These transect walks enabled general observation of the land use patterns, vegetation types and distribution, farming methods and mosquito breeding sites.

Mosquito breeding sites were sampled longitudinally using a standard mosquito dipper^1^ [34–36]. The dipping was very carefully made to avoid casting shadow in the habitat as larvae are very sensitive and would dive to the bottom once the shadow is cast on the water. The dipper was lowered gently in an angle of 45° just below the surface so that water flows in together with any larvae that might be present. A total of 211 mosquito habitats were observed. From each habitat a maximum of 6–10 dips were made and sampled larvae were identified based on morphological characteristics, classified into genus level, that is, Anopheles and Culicines [37]. The information obtained here brought insight on the presence of immature mosquitoes in the sites. The investigation on the presence of mosquito breeding site aimed at establishing availability of immature anopheles in relation to location of households and to examine the frequency of contact between mosquitoes and humans.

### Data analysis

Qualitative data from various sources were examined and analysed based on their content while quantitative data were coded and entered in a computer where the STATA software version 10 was used for analysis. Descriptive statistics were run to obtain frequencies, proportions and cross-tabulations. Chi-square test was conducted to establish the strength of relationships between the variables under study. Cross-tabulation allowed a comparison of different study parameters. Tables and graphs were used to present different variables.

## Results

### Socio-demographic characteristics of the respondents

The ages of a large proportion (50.4%) of the respondents ranged between 18 and 35 years (Table 1). The age between 36 and 55 years comprised of 11.7% male and 19.1% female respondents, while those above 55 years constituted of 8.6% males and 10.4% females. The age distribution is a reflection of the variation in the experiences regarding land use patterns, malaria transmissions and control. However, the relationship between age group and factors influencing presence of mosquitoes within homesteads was insignificant.

**Table 1:** Age of respondents expressed in percent

Age group (years)	Kimamba A		Kimamba B		Total	
	Male	Female	Male	Female	Male	Female
18–35	21.3	38.4	18.5	22.5	19.9	30.5
36–55	11.2	12.9	12.1	25.2	11.7	19.1
56+	6.4	9.8	10.7	11	8.6	10.4
Total	38.9	61.1	41.3	58.7	40.1	59.9

Majority of the inhabitants (62.5%) attained primary education (16.6% males and 45.9% females), while about one-third (32.3%) of the respondents never went to school (7.5% males; 24.8% females). Only 4.8 and 0.5% of respondents attended secondary and post-secondary education, respectively (Table 2). Although more educated communities are expected to have better understanding of the modes of malaria transmission and prevention, the analysis of the relationship between education level and malaria knowledge was statistically insignificant possibly because of the generally low knowledge of mosquito ecology and breeding habitats as indicated by respondents in this study.

**Table 2:** Education level of respondents expressed in percent

Level of education	Kimamba A		Kimamba B		Total	
	Men	Women	Men	Women	Male	Female
None	9.5	27.3	5.5	22.3	7.5	24.8
Primary	15.9	41.8	17.2	50.0	16.6	45.9
Secondary	1.0	4.5	1.5	2.5	1.3	3.5
Post-secondary education	0	0	1.0	0	0.5	0
Total	26.4	73.6	25.2	74.8	25.8	74.2

The findings showed that 91.1 and 3.9% of communities in Kimamba depended largely on crop cultivation and small businesses respectively (Table 3). The major crops grown in the area included maize, paddy, beans, cassava, sweet potatoes, cotton, sunflower, sesame and sisal. Other occupations such as employees, pastoralism and mixed farming were reported by only small proportions of respondents. About 3.3% reported to be involved in small businesses activities such as bicycle mechanic, making cook stoves, carpentry and food vending. This diversity of activities has a meaning to the local economies and livelihoods as it provides households with some extra income and enhances livelihood security.

**Table 3:** Major economic activities of the respondents expressed in percent

Occupation	Kimamba A	Kimamba B	Average
Crop cultivation	94.3	87.8	91.1
Petty trade/Business	2.4	5.3	3.9
Employees	0	2.7	1.4
Pastoralism	0.5	0	0.3
Mixed farming	0	0.5	0.3
Others	2.8	3.7	3.3
Total	100	100	100

Agricultural practices such as irrigation of vegetable gardens, maize and rice farms provided suitable breeding grounds for mosquitoes. However, it was found that farmers’ knowledge about malaria and agricultural practices which favour the breeding of mosquitoes was low.

### Land use patterns associated with malaria transmission

**Fig. 2:**
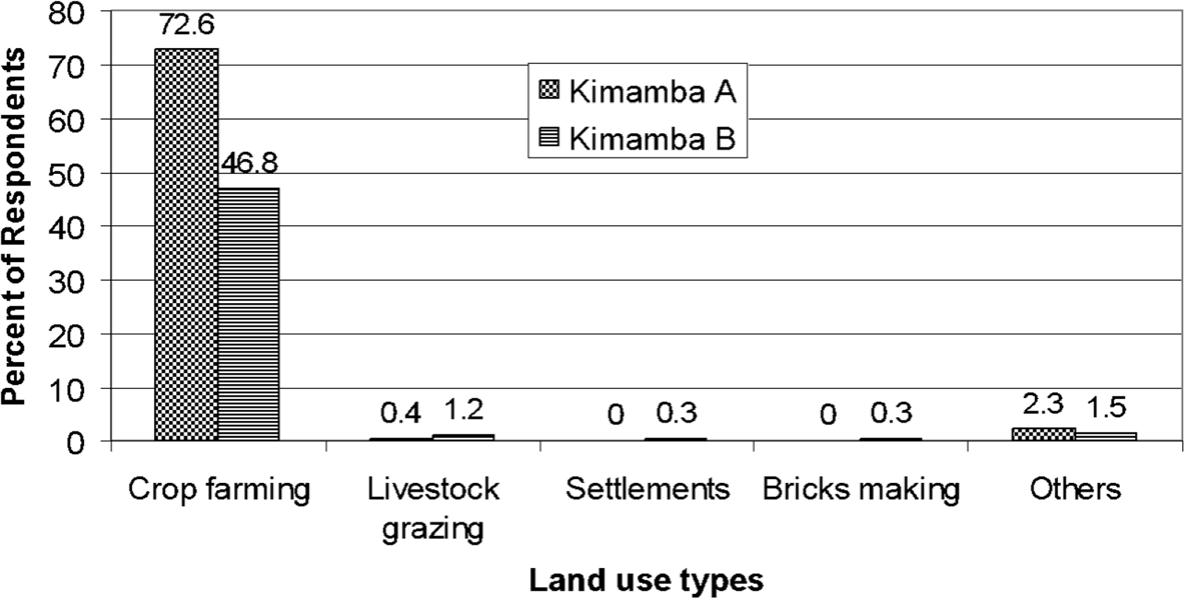
Main land uses in Kimamba Villages

Major land use types found in Kimamba included settlement, crop farming, brick making and livestock grazing (Fig. 2). Crop farming was the main economic activity in Kimamba. Majority (91%) of the respondents reported to be engaged in crop production. The major food crops grown were rice, maize, beans, cassava and plantains while the major cash crop was sisal. However, rice, maize and beans production was both for food and cash. Kimamba A had the highest percentage of people engaged in crop farming as compared to Kimamba B. Only a few (0.3%) of respondents reported to be engaged in livestock keeping, which includes cattle, goats and sheep. The findings of this study compare well with the district, regional and national statistics that indicate that majority of Tanzanians (over 80%) are employed in the agricultural sector [38].

The large part of land in Kimamba villages is still under sisal estates (Fig. 1). The inhabitants of Kimamba and the nearby villages were and some are still employees and labourers of the sisal estates. The inhabitants are only left with very small land for cultivation and other few areas left for settlement and house construction. It was reported by almost all respondents that for them to cultivate they have to either rent a piece of land from the investors for 2000/= per acre or purchase land in nearby villages located between 4 and 18 km from the respondents’ homesteads, which takes 1–4 h walk. As they go to their farms most farmers prefer to stay overnight rather than returning home every day. The mentioned reasons of staying overnight included reducing travel times to and from the field as reported by 16% of respondents, protecting crops from grazing domestic animals (10%), and protecting crops from wild animals (11%). Other reasons given included farm preparation (7%), planting (8%), weeding (13%) and during harvesting season (51%) when much time is needed in the field. It is during such times when they are bitten by mosquitoes.

The study finding indicated that 64.9% of respondents suffered from malaria during the long rainy season (April to June). This is the season when people spend most of their time in their farms by temporarily shifting from their houses to farm shelters. However, about 78% of respondents reported to take with them mosquito nets to use while they are in their farm shelters to protect themselves from mosquito bites. The relationship between use of mosquito nets and its effectiveness in preventing malaria was not significant at 5% (p > 0.05). This was possibly due to the fact that, the mosquito nets were used only for a very short time (during sleeping), while most of the time they are exposed to mosquito bites. Moreover, the poorly built thatch grasses shelters in use have holes and uncovered windows allowing mosquito entry. Other factors include inconsistency of mosquito net use among those who own nets. It was observed that use of nets was interrupted by temporary, periodic or infrequent conditions, which inhibited net use even among regular net users. These conditions included night work, attending late-night social events, disruption of usual sleeping arrangements, net unavailability due to washing or dirtiness, extreme fatigue or forgetfulness [39].

### Brick making and malaria transmissions

**Fig. 3:**
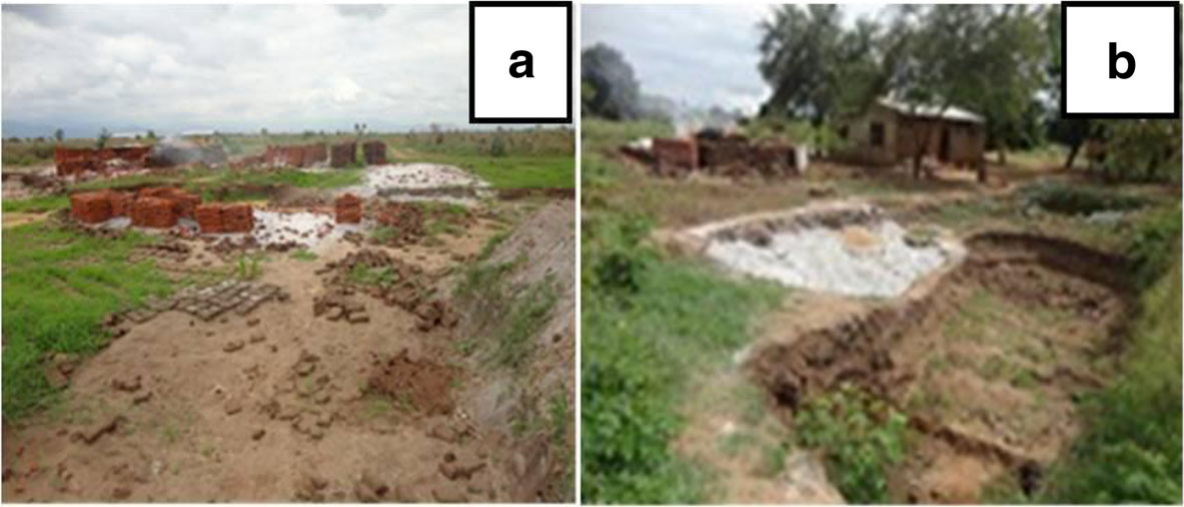
Bricks making sites (**a**) in a public place and (**b**) near the homestead

The study found that these villages had many sites used for brick making and some of them were located around homesteads (Fig. 3). Inhabitants reported to make bricks mostly during the dry season and is normally for business purposes and own house construction. The brick making pits have also been found to increase the outbreak of malaria in highlands of Kenya [40]. Despite many people being involved in brick making, only 0.3% of respondents in the present study knew the close relationship between malaria and brick making.

### Diversity of mosquito breeding sites in Kimamba village

**Fig. 4:**
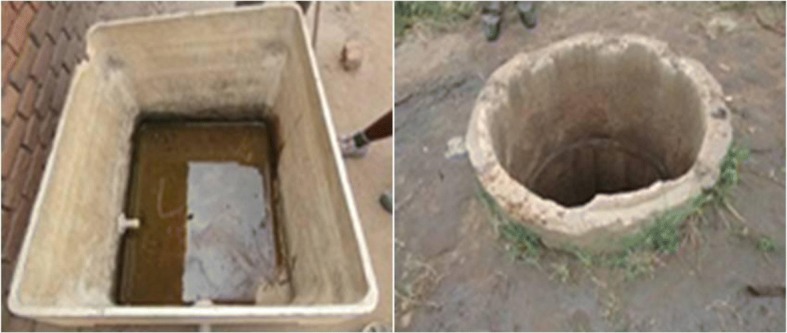
Water container (left) and an open well (right)

**Fig. 5:**
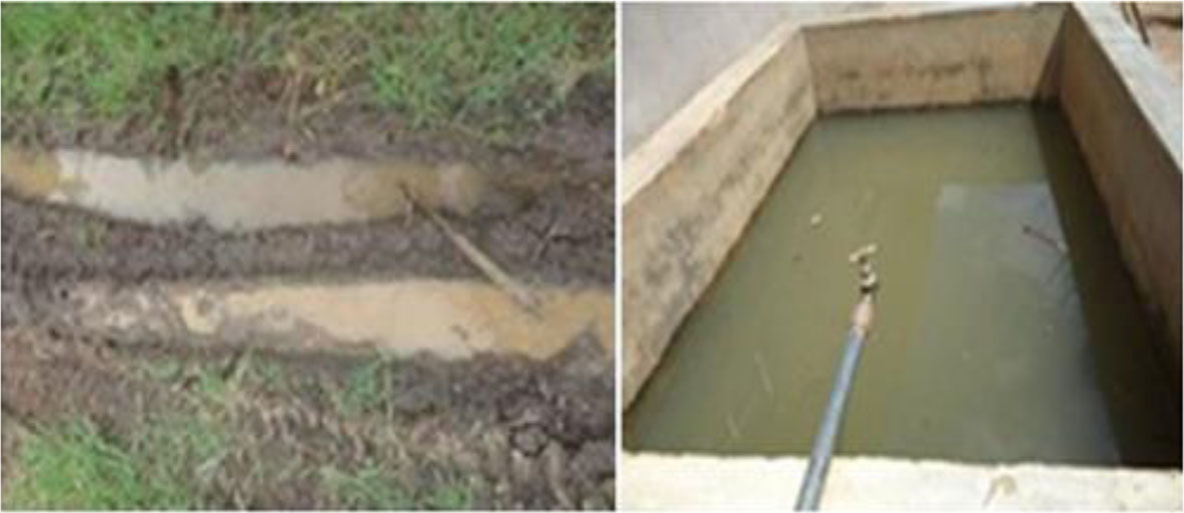
Tyre marks (left) and water tank around homesteads (right)

A total of 211 mosquito habitats (sites) were found around the homesteads (cf. Figs. 4 and 5). These habitats were grouped as small which included hoof prints, coconut shells, tins, broken pots, buckets and tyre marks (Fig. 5); medium habitats included ditches, concrete holes, wells, water tanks, water pools, broken drums streams; and the large habitats were swamps. The findings indicated that 30% of mosquito habitats observed were small, medium water pools (Table 4). Majority of these water pools were due to the brick making activities. When asked what were the main agricultural practices associated with malaria transmissions, most respondents mentioned vegetable gardening, maize and rice cultivation, both rain fed and irrigated.

**Table 4:** Diversity of mosquito breeding grounds in Kimamba villages

Habitat type	Kimamba “A”		Kimamba “B”			Total	
	Posta	Uhindini	Mkwajuni	Sikutali	Sokomsuya	Number	%
Water pools	10	5	21	25	3	64	30
Wells	1	2	5	14	2	24	11
Broken pots	7	0	8	7	0	22	10.4
Hoof prints	0	14	0	8	0	22	10.4
Tins	5	2	8	4	0	19	10
Coconut shells	0	0	6	11	1	18	8.1
Ditches	6	3	1	3	0	13	6
Broken buckets	0	2	2	5	0	9	3.3
Tyre marks	2	0	0	2	0	4	1.9
Concrete holes	1	1	0	2	0	4	1.9
Broken Drums	0	1	2	2	0	5	2
Old Tyres	1	1	0	0	0	2	1
Banana leaves	2	0	0	0	0	2	1
Swamps	1	0	0	0	0	1	1
Streams	1	0	0	0	0	1	1
Water holes	0	1	0	0	0	1	1
**Total**	37	32	53	83	6	211	100

Mosquito breeding habitats ranged from as small as hoof prints, coconut shells to the large ones such as swamps. Majority (95%) of the observed habitats represent mosquito breeding sites that were man-made. Most of the man-made habitats were linked to livelihood activities such as farming and house construction. The status of the observed mosquito habitats was analyzed and grouped as presented in the Table 5.

**Table 5:** Percent response on the status of mosquito breeding sites in Kimamba village

Villages	Sub-village	Water status		Origin		Associated livelihoods	
		Water	Dry	Man-made	Natural	Farming	Constructions
Kimamba “A”	Posta	9.0	10.4	18.5	0.9	4.7	14.7
	Uhindini	7.1	8.1	12.3	2.8	1.9	13.3
Kimamba “B”	Mkwajuni	17.1	7.1	24.2	0	0.5	23.7
	Sikutali	26.5	12.8	38.4	0.9	14.7	24.6
	Sokomsuya	1.4	0.5	1.9	0	0.5	1.4
	Total	61.1	38.9	95.3	4.7	22.3	77.7

Mosquito larval search was conducted in all breeding sites identified in the study area. Findings showed that 28% out of 61.1% of the total wet mosquito breeding sites identified had immature mosquitoes, while 10 and 8% of these were identified to be Anophelines and Culicines, respectively. The abundance of immature mosquitoes for the two species was associated with ecological factors such as rainfall and breeding habitats.

## Discussion

While the general knowledge that link mosquito and malaria among farming communities in Kilosa district was reported to be high, there is very low knowledge on mosquito ecology and breeding sites among the inhabitants. Such ignorance of the ecology of malaria vector in the study area is likely to increase the mosquito productivity by creating more breeding sites. Agriculture was mostly reported by the respondents as only the reasons for increased malaria intensity in Kimamba villages, because it supports breeding of mosquitoes that carry the malaria parasites. These results are supported by various works done in Tanzania [41–44]. Malaria is a particular problem in agricultural areas, as land use practices implemented often result in increased presence of breeding sites [6, 45]. The influence of agricultural systems on health is particularly notable via the intermediary process of land use change. Agricultural production systems including farming practices, location of farms, and farming technologies could lead to land use change that create suitable ecological and climatic conditions for the breeding and survival of the Anopheline mosquitoes, which transmit the malaria [6].

Despite of the fact that brick making and settlement were highly practiced in Kimamba, the respondents did not directly link them with an increase of malaria productivity. The study by Carlson [40] in western Kenya revealed that brick-makers make bricks predominantly during the dry-season. The whole process of brick making leaves behind temporary and permanent pools which store water especially during rainy seasons. During long rains the number of immature mosquitoes increases while during dry season decreases due to the availability of more breeding sites in the former than latter period. These results conform to the findings from other studies on the population dynamics of the mosquitoes.

Majority of the breeding sites found in this study were created by human. The diversity of breeding sites suggests the possibilities of emergence and re-emergence of malaria as they provide conducive environment that favour mosquito survival. It is common that mosquitoes breed and multiply easily in the temporary and permanent habitats occupied by water with favourable temperature [24, 46]. This study found that majority of the mosquito breeding habitats were located within the homesteads. Presence of these habitats around homesteads increased the contact between humans and mosquitoes hence enhanced malaria transmissions.

Most of the Kimamba inhabitants are involved in temporal and spatial movements, including circulation and migration. Circulation encompasses a variety of movements, usually short-term and cyclical and involving no longstanding change of residence while migration involves a permanent change of residence [47]. It was reported by key informants and households interviewed during the present study that there were seasonal movements of people in the study area. During the farming season people move from various parts of Tanzania to Kimamba where they rent houses and land for farming purposes. Movements from Kimamba villages to their distant farms were also reported. The farming season begins during the short rainfall of October to December. It is quite possible that some people come to this area already infected by malaria or carrying the parasite. The spatial movement to and from malaria areas are of epidemiologic importance. Pim and Lisbeth [47] reported for instance that people who move can be categorized as either active transmitters or passive acquirers. Active transmitters harbour the parasite and transmit the disease when they move to areas of low or sporadic transmission. Passive acquirers are exposed to the disease through movement from one environment to another; they may have low-level of immunity or may be non-immune, which increases their risk for malaria. Similar experiences were reported for highland areas of southern Tanzania by Kangalawe [19].

During farm activities people shift temporarily from their traditional homesteads to distant farms. Thus, it is possible that during this shift people transmit malaria from the farms where they mostly contract the disease to their permanent areas [47]. The University of Richmond [48] maintained that humans become vectors capable of transmission when they become active transmitters. According to Pim and Lisbeth [47], active transmitters harbour the parasite and transmit the malaria when they move to areas of low or sporadic transmission. Passive acquirers become active transmitters once they are infected because humans can transmit malaria gametocytes to uninfected mosquitoes or mosquitoes infected with asexual (non-infective) stage of Plasmodium development [49].

## Conclusion

This study shows that changes in land use patterns are associated with malaria transmission as demonstrated by presence of diverse habitats for mosquitoes in the land units used for various purposes. Although the analysis of land use patterns did not show statistical significance in the study area, agricultural activities, brick making and settlement seem to be highly linked to the malaria transmission. The association of land use patterns and malaria transmission is well observed in habitats created that harbour mosquitoes, and evidenced by presence of immature of Anopheles and Culicines mosquito larvae. The presence of diverse habitats in various land use patters suggests that controlling malaria transmission in those areas would need multi-stakeholder, multidisciplinary and integrated approaches. Lack of knowledge of the epidemiology of transmission by the inhabitants is a major issue. Although it might be difficult to change land use patterns, as they are driven by economic necessity, future reduction of spread, through better education, is something that could be modified. In addition, more detailed studies are recommended to further confirm the linkages between land use/cover changes and malaria transmission in the study area.

## Abbreviations

FAO: Food and Agriculture Organization
IPMA: Integrated Research Partnership for Malaria - Eco-Health Approach
NIMR: National Institute for Medical Research
URT: United Republic of Tanzania
X^2^: Chi-Square

## Footnotes

1 The standard mosquito dipper is the most commonly used tool for collecting mosquito larvae and pupae from a wide variety of habitats. Dippers vary in size and shape but are often white to facilitate detection of larvae. Local availability often dictates the size of dipper used, which unfortunately is not always clearly recorded with the results of surveys.
